# Lanthanide(III) and Yttrium(III) Complexes of Benzimidazole-2-Acetic Acid: Synthesis, Characterisation and Effect of La(III) Complex on Germination of Wheat

**DOI:** 10.1155/BCA/2006/75612

**Published:** 2006-03-23

**Authors:** Kalagouda B. Gudasi, Rashmi V. Shenoy, Ramesh S. Vadavi, Manjula S. Patil, Siddappa A. Patil, Rayappa R. Hanchinal, Srinivas A. Desai, H. Lohithaswa

**Affiliations:** ^1^Department of Chemistry, Karnatak University, Dharwad 580 003, Karnataka, India; ^2^University of Agricultural Sciences, Dharwad 580 005, Karnataka, India

## Abstract

The synthesis and characterisation of lanthanide(III) and yttrium(III) nitrate complexes of benzimidazole-2-acetic acid (HBIA) are reported. The complexes have been characterised by elemental analysis, molar conductance, magnetic studies, IR, ^1^H NMR, UV-visible, EPR, and TG/DTA studies. They have the stoichiometry [Ln_3_(BIA)_2_(NO_3_)_7_(H_2_O)_4_] · 3H_2_O where Ln=La(III), Pr(III), Nd(II), Sm(III), Eu(III), Gd(III), Tb(III), Dy(III), and Y(III). The effect of La(III) complex on germination, coleoptile, and root length of two local varieties of wheat DWR-195 and GW-349 for different treatment periods has been investigated. The complex was found to exhibit enhanced activity, compared to HBIA or metal salt alone at lower treatment periods.

## INTRODUCTION

The use of rare earth elements (REE) in agriculture to promote the growth of plants is well known [1–4]. The utilization of REE containing fertilizers is widespread in China due to its rich REE resources. They are found to have nitrogen-fixing capacity, to enhance seed germination, strengthen photosynthetic rate, to enhance respiration and activity of hydrolytic enzymes and plant hormones, and reduce water loss [5, 6]. Chen et al [7] have reported the stimulation in growth of tobacco seedling treated with lanthanide. Enhancement in the germination of lanthanide-treated aged rice seeds has been reported by Fashui [8].

Heterocyclic compounds like indole-3-acetic acid and naphthyl-1-acetic acid are the plant auxins found to affect the growth of plants [8]. Especially benzimidazole-containing heterocyclic moieties have found extensive use in agriculture [9]. The lanthanide complexes of a few selected compounds have exhibited fungicidal and bactericidal activities including regulating the growth of plants [10–14].

It is known that chelation of metal ions with organic ligands acts synergistically to increase its effect [15].

Keeping this in view, the Ln(III) and Y(III) complexes of benzimidazole-2-acetic acid (HBIA) have been synthesized and characterised. La(III) complex of HBIA was used to carry out the germination study. The present experiment was conducted with a view to study the following.

1. Effect of complex on germination, coleoptile, and root length of wheat.
2. Comparative effect of complex, ligand, salt, and meth-anol on the above-mentioned factors compared to the control.

Lanthanides at high concentration are toxic whereas lower concentrations promote plant growth action, called the Hormesis effect [16]. Hence wheat seeds were treated with 10^−6^ M solution for different treatment periods.

## EXPERIMENTAL

### Chemicals and instruments

Reagent grade chemicals were used without further purification. Ethyl cyanoacetate (S. D. Fine Chem Ltd, India) and o-phenylenediamine (Koch Light Laboratories Ltd., England) was used. The metal contents of the complexes were determined by complexometric titrations against EDTA. Carbon, hydrogen, and nitrogen contents were determined by using a Carlo-Erba Strumentazione (Italy) CHN analyzer. Molar conductivities in DMSO (10^−3^ M) at room temperature (26°C) were measured using an Elico conductivity bridge having platinum electrodes. Magnetic moments were determined by a Faraday balance. The IR spectra of ligand and its metal complexes were recorded on a Nicolet 170 SX FT-IR spectrometer in the range 400–4000 cm^−1^ using KBr discs. The EPR spectrum of the Gd(III) complex was recorded on a Varian E-4X band spectrophotometer. ^1^H NMR spectra were recorded on a Bruker Avance 300 MHz NMR spectrometer using DMSO-d_6_ as solvent. UV-visible spectra were measured on a Hitachi 2001 spectrophotometer using dimethylsulfoxide (DMSO) as solvent. Thermogravimetry (TG) and differential thermal analysis (DTA) measurements were made in N_2_ atmosphere between 20 and 1000°C using a Perkin-Elmer (Pyris Diamond) analyzer. Lanthanide nitrates were prepared by dissolving the corresponding oxide (99.99%, Indian Rare Earths Ltd, India) in 50% HNO_3_, followed by the evaporation of the excess acid.

#### Synthesis of ligand

The ligand was synthesized according to literature method [17].

#### (a)Preparation of cyanomethyl benzimidazole

**Scheme 1:**
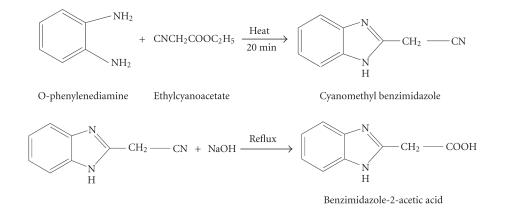
Preparation of benzimidazole-2-acetic acid.

o-phenylenediamine (10 mmol, 10.0 g) and ethylcyanoacetate (15 mmol, 17 g) were placed in the reaction tube and heated in boiling aniline for 20 minutes. The residue was broken up and extracted with ether. The residue was recrystallised from hot water with the aid of norite and finally from alcohol and water (Scheme 1).

- Yield: 11 g (70%),
- MP: 209.7–210.7°C.

#### (b)Alkaline hydrolysis

2-cyanomethyl benzimidazole (10 mmol, 1.57 g) was added to 20 cc of a water solution containing 1.2 g of NaOH and 3 cc of ethanol and refluxed for 2 hours or until no more ammonia was evolved. The solution was then acidified with acetic acid.

- Yield: 1.65 g (95%),
- MP: 116°C (with decarboxylation, solidification, and remelting at 176°C (2-methyl benzimidazole)).

#### Synthesis of complexes

The ligand was first converted into its sodium salt by refluxing NaOH (2 mmol, 0.080 g) and HBIA (2 mmol, 0.0352 g) in 20 ml methanol for half an hour. Ln(NO_3_)_3_ (1 mmol) dissolved in minimum amount of methanol was added and further refluxed for 3 hours. The solution was then concentrated to a small volume and the precipitate obtained was filtered, washed with water and ethanol, and air-dried.

- Yield: 75%.

## MATERIALS AND METHODS

### Procedure

Two local varieties of wheat (DWR-195 and GW-349, developed at UAS, Dharwad) were selected for the experiment. The solution of compounds (10^−6^ M) was prepared in methanol due to its insolubility in water. The compounds to be tested consisted of La(BIA) complex, HBIA ligand, La(NO_3_)_3_, methanol, and water (control) assigned as groups 1, 2, 3, 4, and 5, respectively. The periods of treatment varied from 2, 5, 8, and 11 minutes assigned as T1, T2, T3, and T4, respectively.

The seeds were soaked in the respective solutions for the above-mentioned time periods and then placed between germination papers (46 cm × 29 cm in size) previously soaked with water in two rows. They were then rolled carefully ensuring no excess pressure was placed on seeds, wrapped in a sheet of polythene film to reduce surface evaporation, and placed in a germination chamber. On the fifth day after germination, the germination count was taken. Ten seedlings (out of 25 taken) were randomly selected and measured for their coleoptile and root length. Three replications were carried out for each observation.

### Statistical analysis

The statistical evaluation of the results was conducted with use of SPSS-(statistical package for social science; Windows version 6.0) packed program. One-way analysis of variance (ANOVA) was used to analyze the results. The level of statistical significance was set at *P* < .05.

## RESULTS AND DISCUSSION

### Germination percentage

In case of variety GW-349 (Table 1), there was no significant difference between all the groups at T1 and T2. In case of T3, groups 3 and 4 showed significant decrease in germination percentage (GP) compared to the control (*P* < .05). At T4, group 5 showed significant increase in GP compared to groups 2, 3, and 4. Group 1 showed significant increase in GP compared to groups 3 and 4.

In case of variety DWR-195 (Table 2), at T1, T2, and T3 there was no significant difference in GP in case of all the groups. At T4, group 1 seeds showed significant increase in GP compared to group 5(control).

### Root length

In case of variety GW-349 (Table 3), at T1, group 1 showed significant increase in root length (RL) compared to groups 2, 4, and 5(control). At T2, T3, and T4 there was no significant differences between the groups.

In case of variety DWR-195 (Table 4), at T1, T2, and T4, group 1 shows significant increase in RL compared to groups 2, 3, 4, and 5(control), while at T3 group 1 does not show any significant differences between the groups.

### Shoot length

In case of variety GW-349 (Table 5), group 1 showed increased shoot lengths (SL) compared to groups 2, 4, and 5 at T1, T2, and T3 while group 3 showed significant difference compared to group 1. At T2 and T3, group 1 showed increased SL compared to group 3. However, at T3 and T4, group 1 showed no significant increase in SL compared to control, and compared to group 2 at T4. Group 1 showed significant increase in SL compared to groups 3 and 4 at T4.

In case of variety DWR-195 (Table 6), group 1 showed significant increase in SL for the treatment periods T1, T2, T3, and T4 compared to groups 2, 3, 4, and control. However group 3 showed significant increase in SL compared to group 1 at T3 while at T4 there was no significant difference between group 1 and control.

### General aspects

The complexes are nonhygroscopic, off-white in color, and have 3 : 2 (M : L) stoichiometry (Table 7). The complexes are almost soluble in methanol, ethanol and completely soluble in DMF and DMSO whereas they are insoluble in acetone, chloroform, benzene, and ethyl acetate. The molar conductivity values of the complexes in 10^−3^ M DMSO (Table 7) are in the range 4.4−9.6 Ohm^−1^ cm^2^ mol^−1^ indicating their nonelectrolytic nature [18].

Magnetic moment values indicate the absence of metal-metal interaction and the noninvolvement of 4f electrons in bonding.

### Spectral analysis

#### Infrared

The IR spectral data of HBIA and its Ln(III) complexes is given in Table 8. In the free ligand, a broad band in the region 2525–3391 cm^−1^ is due to intramolecular hydrogen bonding between OH of carboxylic group and C=N [19]. The bands at 1647 cm^−1^ and 1613 cm^−1^ are assigned to the *ν*(C=O) and *ν*(C=N) groups, respectively [19]. The −NH stretching frequency is observed at 3164 cm^−1^. An intense band observed at 1567 cm^−1^ is characteristic of 2-substituted benzimidazoles [20].

In the complexes, the *ν*NH vibrations appear in the range 3048–3055 cm^−1^ and has a comparatively lower value compared to that for the free ligand. On complexation, the *ν*C=O frequency vanishes and is replaced by two bands between 1585–1591 cm^−1^ and 1431–1442 cm^−1^ assignable to *ν*_asy_(COO) and *ν*_sym_(COO), respectively. In the spectra of sodium salt of the ligand, the *ν*_asy_(COO) and *ν*_sym_(COO) occur at 1586 and 1441 cm^−1^, respectively.

In the present complexes, the difference between the *ν*_asym_(COO) and *ν*_sym_(COO) lies in the range 148–160 cm^−1^ whereas that of the sodium salt is 145 cm^−1^ indicating the coordination of carboxylate group to metal ions in bidentate bridging fashion [21, 22]. The *ν*(C=N) band is shifted to lower frequency merging with the *ν*_asym_(COO) band indicating its involvement in coordination.

The presence of coordinated nitrate groups is confirmed by the presence of six fundamental bands at 1313, 1525, 739, 708, 998, and 850 cm^−1^ which are assigned to *ν*_1_, *ν*_4_, *ν*_3_, *ν*_5_, *ν*_2_, and *ν*_6_, respectively. The difference between the two highest bands *ν*_4_ and *ν*_1_ lies in the range 206–216 cm^−1^, indicating the coordination of nitrate groups in bidentate or bidentate chelating fashion [23, 24].

A strong and broad absorption between 3375–3394 cm^−1^ in the complexes indicates the presence of water molecules.

#### ^1^H NMR spectra

**Figure 1:**
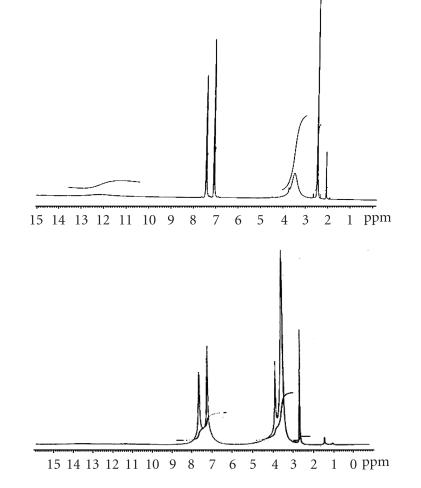
^1^H NMR spectra of HBIA and La(III) complex.

The ^1^H NMR spectra of HBIA and its La(III) complex (Figure 1) was carried out in DMSO-d_6_. The proton assignments are given in Table 9.

A broad signal at 12.20 ppm in the “free” ligand is ascribed to −OH of the carboxylic group which is intramolecularly hydrogen-bonded with the nitrogen of the benzimidazole moiety. Two quartets at 7.10 and 7.45 ppm is due to four aromatic protons of the benzene moiety. A broad singlet at 3.5 ppm is due to NH. A sharp singlet at 2.5 ppm is assignable to two protons of the −CH_2_ group which also overlaps with the DMSO peak.

On complexation, the −OH signal disappears completely indicating the breakdown of intramolecular hydrogen bonding along with ligation of COO− to metal ion. The downfield shift of the −NH signal from 3.5–3.7 ppm indicates the involvement of *ν*(C=N) in coordination with the metal ion. The −CH_2_ proton undergoes a very slight upfield shift to 2.48 ppm. The aromatic protons appear in the region 7.07–7.47 ppm.

#### UV-visible spectra

The electronic spectra of HBIA displays three absorption bands at 276, 270, and 257 nm which are assigned to the *π*-π* transitions. The electronic spectra of the complexes are similar to that of ligand except for a very slight shift towards lower frequency. A comparison of the transitions of the complexes with their corresponding aquo ions has been made (Table 10). This data clearly indicates that the energy of f-f transitions in the complexes is slightly reduced from the corresponding aquo ions, due to perhaps covalent interaction of 4f orbitals with vacant ligand orbitals or increased nuclear shielding of f-orbitals due to slight L-M electron transfer [25]. The nephelauxetic ratio (*β*), bonding parameter (*b* ^1/2^), Sinha's covalency parameter (δ%), and angular covalency (*η*) for the Pr(III) and Nd(III) complexes have been calculated [26–29].

The Sinha parameter (*δ*) is taken as a measure of covalency and is given by

(1) $$\delta (\%)=\frac{1-\beta_{\text{av}}}{\beta_{\text{av}}}\times 100$$,

where *β*_av_ is the average value of the ratio of *ν*_complex_/*ν*_aquo_.

The bonding parameter (*b* ^1/2^), the magnitude of which suggests the comparative involvement of the 4f orbitals in metal-ligand bonding, is related to the nephelauxetic ratio *β* and is given by the expression

(2) b12=[1−βav2]12.

δ values are less than one whereas the remaining values are positive indicating the interaction between the metal salts and ligand is mainly electrostatic.

#### EPR spectra

**Figure 2:**
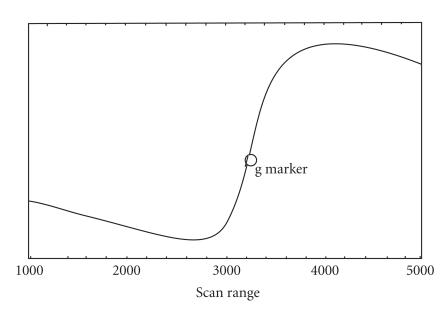
EPR spectrum of [Gd_3_(BIA)_2_(NO_3_)_7_(H_2_O)_4_] · 3H_2_O (at RT).

The EPR spectra of the Gd(III) complex (Figure 2) showed broad resonance peaks with g = 1.99 (at RT) and 1.97 (at LNT). Similar g values and line widths (at both RT and LNT) indicate that line widths are independent of temperature [30]. It is observed that fine structure, as a consequence of Gd(III) zero-field splitting is lacking. The broad peaks obtained indicate that the Gd(III) complex is located in a rather disordered environment. Thus the spectrum is an average overall possible realization of the crystal field, which can be influenced by distribution of hydrogen bonds. Random H-bonds between water molecules and complex induce small distortions, which lead to line broadenings. This phenomenon called strain, more specifically g-strain for the g-tensor distribution and D-strain for the ZFS (zero-field splitting) distribution, leads to broad asymmetric EPR line shapes [31, 32].

#### Thermal analysis

**Figure 3:**
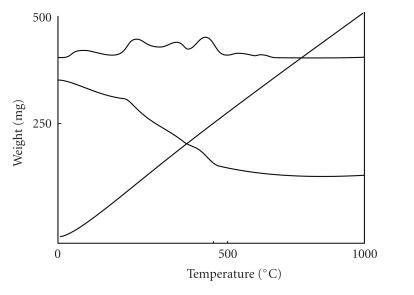
TG/DTA thermogram of [Dy_3_(BIA)_2_(NO_3_)_7_(H_2_O)_4_] · 3H_2_O complex.

The TG/DTA study of a representative Dy(III) complex (Figure 3) was determined in nitrogen atmosphere up to 1000°C using a heating rate of 10°C/min.

The weight loss of 3.64% on TG curve between 29–79°C reveals the loss of three-lattice-held water molecules, which is in agreement with the calculated values of 3.85%. The next weight loss of 5.09% between 80–150°C agrees (5.14%) with the loss of four coordinated water molecules. A weight loss corresponds to the loss of a ligand molecule and four coordinated nitrate molecules (calc: 30.27%), in the temperature range 280–540°C. Above 500°C there is a plateau, which corresponds to the formation of stable Dy_2_O_3_. The metal percentage obtained is 34.78%, which agrees well with calculated value (34.83%) and is within the experimental error.

**Figure 4:**
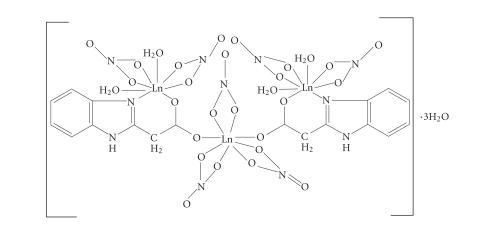


The tentative structure was arrived at on the basis of the various spectral studies and is shown in Figure 4.

## CONCLUSION

The results obtained in the present study indicate that in both the varieties, *germination percentage* of water-treated seeds and complex treated seeds was not significantly different at lower treatment periods of 2, 5, and 8 minutes indicating germination is not affected by the treatments. In case of *root length* the root lengths of complex-treated seeds, at lower treatment period of 2 minutes, were significantly higher than the water-treated ones.

In case of *shoot length*, in both the varieties the complex-treated seeds showed significant increase in shoot length compared to the water-treated ones at lower treatment periods of 2, 5, and 8 minutes.

In all the cases, the complex-treated seeds showed significant increase compared to those treated with ligand and metal salt alone.

From this, it is concluded that the La(NO_3_)_3_ salt or HBIA alone is not effective but the complex acts synergistically to enhance the effect of the two. In case of period of treatments, lower treatment period (2 minutes) was found to be most effective in all the cases.

Hence lanthanide complexes of benzimidazoles and other auxins may prove to be very good plant growth promoters.

## Figures and Tables

**Table 1:** Effect of La(III) complex on germination of wheat (variety GW-349).

Groups	Treatments			

	T1 (2 min)	T2 (5 min)	T3 (8 min)	T4 (11 min)

Group1 La(III) complex	98.66	97.33	96.66	96.66
Group 2 (BIA ligand)	95.0	94.0	93.0	91.33
Group 3 La(NO_3_)_3_ salt	94.0	95.0	91.33	89.66
Group 4 methanol	94.0	92.33	91.33	89.66
Group 5 control (water)	97.33	98.66	98.66	100

**Table 2:** Effect of La(III) complex on germination of wheat (variety DWR-195).

Groups	Treatments			

	T1	T2	T3	T4

Group1	97.33	95.0	95.0	94.0
Group 2	95.0	96.0	94.0	94.0
Group 3	95.0	93.0	93.0	89.66
Group 4	95.0	94.0	91.33	91.33
Group 5	95.66	95.33	96.0	98.66

**Table 3:** Effect of La(III) complex on root length (in mm) of wheat (variety GW-349).

Groups*	Treatments*			

	T1	T2	T3	T4

Group1	134.86	126.83	110.26	120.66
Group 2	116.56	116.03	111.93	110.56
Group 3	114.83	107.93	101.96	105.46
Group 4	111.50	114.70	100.80	104.0
Group 5	113.36	115.80	123.83	123.23

*Please refer to Table 1 for details of treatments and groups.

**Table 4:** Effect of La(III) complex on root length of wheat (in mm) (variety DWR-195).

Groups*	Treatments*			

	T1	T2	T3	T4

Group1	138.70	135.03	125.10	135.66
Group 2	153.86	132.30	123.0	101.80
Group 3	121.96	124.56	123.76	123.46
Group 4	129.16	127.56	126.23	121.66
Group 5	129.0	123.53	122.86	131.83

*Please refer to Table 1 for details of treatments and groups.

**Table 5:** Effect of La(III) complex on shoot length (in mm) of wheat (variety GW-349).

Groups*	Treatments*			

	T1	T2	T3	T4

Group1	72.76	68.06	65.26	62.53
Group 2	67.70	53.30	61.26	63.26
Group 3	75.0	52.03	52.33	48.93
Group 4	57.96	60.36	50.96	43.0
Group 5	61.46	63.86	65.70	65.16

*Please refer to Table 1 for details of treatments and groups.

**Table 6:** Effect of La(III) complex on shoot length (in mm)of wheat (variety DWR-195).

Groups*	Treatments*			

	T1	T2	T3	T4

Group1	76.0	69.36	64.63	61.76
Group 2	60.4	58.60	53.63	50.33
Group 3	58.33	61.80	71.10	42.23
Group 4	57.83	56.73	52.53	53.43
Group 5	55.03	61.33	52.30	62.20

*Please refer to Table 1 for details of treatments and groups.

**Table 7:** Elemental analyses, magnetic moment, and conductance data of BIA and its Ln(III) complexes (calculated values are in paren-theses).

Compound	Found (Calcd) (%)				Magnetic^a^ moment	Molar conductivity^b^
	M	C	H	N		

(1) BIA	—	16.29	2.15	11.62	—	—
		(16.25)	(2.10)	(11.59)		
(2) [La_3_(BIA)_2_(NO_3_)_7_(H_2_O)_4_] · 3H_2_O	31.65	16.24	2.11	11.58	dia	7.6
	(31.70)	(16.18)	(2.09)	(1.53)		
(3) [Pr_3_(BIA)_2_(NO_3_)_7_(H_2_O)_4_] · 3H_2_O	32.14	16.16	2.16	11.40	3.55	7.2
	(32.10)	(16.06)	(2.08)	(11.45)		
(4) [Nd_3_(BIA)_2_(NO_3_)_7_(H_2_O)_4_] · 3H_2_O	32.74	15.91	2.13	11.35	3.61	8.3
	(32.61)	(15.84)	(2.05)	(11.29)		
(5) [Sm_3_(BIA)_2_(NO_3_)_7_(H_2_O)_4_] · 3H_2_O	33.42	15.85	2.01	11.19	1.64	6.9
	(33.53)	(15.79)	(2.04)	(11.25)		
(6) [Eu_3_(BIA)_2_(NO_3_)_7_(H_2_O)_4_] · 3H_2_O	34.69	15.68	2.07	11.17	3.40	7.7
	(34.66)	(15.60)	(2.02)	(11.12)		
(7) [Gd_3_(BIA)_2_(NO_3_)_7_(H_2_O)_4_] · 3H_2_O	34.46	15.66	2.06	11.13	7.79	8.6
	(34.54)	(15.55)	(2.01)	(11.08)		
(8) [Tb_3_(BIA)_2_(NO_3_)_7_(H_2_O)_4_] · 3H_2_O	34.67	15.52	2.01	11.12	9.82	4.4
	(34.77)	(15.43)	(2.0)	(11.0)		
(9) [Dy_3_(BIA)_2_(NO_3_)_7_(H_2_O)_4_] · 3H_2_O	35.21	18.00	2.25	13.17	10.30	9.6
	(35.28)	(18.32)	(2.37)	(13.06)		
(10) [Y_3_(BIA)_2_(NO_3_)_7_(H_2_O)_4_] · 3H_2_O	23.01	47.16	3.49	18.70	dia	8.0
	(22.97)	(47.06)	(3.50)	(18.78)		

^a^BM. ^b^Ohm^−1^ cm^2^ mol^−1^.

**Table 8:** Infrared spectral data of BIA and its Ln(III) complexes (b = broad, s = strong, m = medium, w = weak, BIA^−^Na^+^ = sodium salt of ligand).

Compound	*ν*o_H_	*ν*NH	*ν*C=O	*ν*_asy_COO	*ν*_sym_COO	Δ*ν* = *ν*_asy_COO–*ν*_sym_COO	*ν*_4_	*ν*_1_	*ν*_3_	*ν*_5_	*ν*_6_	*ν*_2_

BIA	3391	3164 s	1647s	—	—	—	—	—	—	—	—	—
(BIA)Na	—	3164s	—	1586s	1441s	145	—	—	—	—	—	—
(2)	3388b	3055s	—	1591sb	1431s	160	1525	1313	739	708	858	998
(3)	3388b	3048s	—	1591sb	1431s	160	1525	1313	745	705	850	997
(4)	3388b	3049s	—	1591sb	1437s	154	1525	1319	745	695	850	998
(5)	3375b	3048s	—	1591sb	1431s	160	1535	1319	745	707	850	998
(6)	3388b	3055s	—	1591sb	1437s	154	1529	1313	739	702	856	998
(7)	3394b	3054s	—	1597sb	1442s	155	1528	1316	742	707	850	999
(8)	3394b	3054s	—	1591sb	1442s	149	1528	1319	745	701	850	996
(9)	3376b	3055s	—	1585sb	1437s	148	1528	1320	745	702	856	996
(10)	3388b	3055s	—	1597sb	1437s	160	1529	1320	745	702	856	998

**Table 9:** ^1^H NMR spectral data (ppm) of BIA and its La(III) complex (b = broad; q = quartet; d = doublet; s = singlet).

Proton	BIA	La(III) complex

−OH	12.20 (s, br)	—
−NH	3.5 (s, br)	3.70 (s)
−CH_2_	2.5 (s)	2.48 (s)
Aromatic protons	7.45 (q, 2H)	7.47 (d, 2H)
—	7.10 (q, 2H)	7.07 (d, 2H)

**Table 10:** Electronic spectral data of few Ln(III) complexes of BIA.

Complex	Assignments	*λ*_max_ of Ln^+3^	*λ*_max_ of complex	*β*	Other parameters
		ion(cm^−1^)	(cm^−1^)		

(1) [Pr_3_(BIA)_2_(NO_3_)_7_(H_2_O)_4_] 3H_2_O	^3^H_4_- - -^3^P_2_	22 512	22 502	0.999 55
	- - -^3^P_0_	20 716	20 678	0.998 16	δ = 0.105 11
	- - -^1^D_2_	16 854	16 840	0.999 16	b^1/2^ = 0.022 91
					*η* = 0.032 42
				0.998 95

(2) [Nd_3_(BIA)_2_(NO_3_)_7_(H_2_O)_4_] 3H_2_O	^4^I_9/2_- - -^2^G_9/2_	19 747	19 565	0.990 78
	- - -^4^G_5/2_	17 253	17 182	0.995 88	δ = 0.707 97
	- - -^4^F_9/2_	14 594	14 450	0.990 13	b^1/2^ = 0.0592
	- - -^4^F_5/2_	12 853	12 790	0.995 09	*η* = 0.084 14
			
				0.992 97
